# Role of the Microbiome in Interstitial Lung Diseases

**DOI:** 10.3389/fmed.2021.595522

**Published:** 2021-01-28

**Authors:** Ozioma S. Chioma, Laura E. Hesse, Austin Chapman, Wonder P. Drake

**Affiliations:** ^1^Division of Infectious Diseases, Department of Medicine, Vanderbilt University School of Medicine, Nashville, TN, United States; ^2^Department of Pathology, Microbiology, and Immunology, Vanderbilt University School of Medicine, Nashville, TN, United States

**Keywords:** interstitial lung disease (ILD), fibrosis, sarcoidosis, gut microbiome, infection, idiopathic pulmonary fibrosis, lung microbiome

## Abstract

There are trillions of microorganisms in the human body, consisting of bacteria, viruses, fungi, and archaea; these collectively make up the microbiome. Recent studies suggest that the microbiome may serve as a biomarker for disease, a therapeutic target, or provide an explanation for pathophysiology in lung diseases. Studies describing the impact of the microorganisms found in the respiratory tract on lung health have been published and are discussed here in the context of interstitial lung diseases. Additionally, epidemiological and experimental evidence highlights the importance of cross-talk between the gut microbiota and the lungs, called the gut–lung axis. The gut-lung axis postulates that alterations in gut microbial communities may have a profound effect on lung disease. Dysbiosis in the microbial community of the gut is linked with changes in immune responses, homeostasis in the airways, and inflammatory conditions in the gastrointestinal tract itself. In this review, we summarize studies describing the role of the microbiome in interstitial lung disease and discuss the implications of these findings on the diagnosis and treatment of these diseases. This paper describes the impact of the microbial communities on the pathogenesis of lung diseases by assessing recent original research and identifying remaining gaps in knowledge.

## Introduction

The gut microbiota is defined as the diverse microbial communities that inhabit the host's gastrointestinal (GI) tract (1). The GI tract is a nutrient rich environment that supports up to 100 trillion microbes which collectively make up the gut microbiome (2). The gut microbiome profoundly impacts human physiology and nutrition, and is essential for human life (3). Many factors, including diet, age, antibiotics, lifestyle behaviors, and mode of delivery at birth are influential contributors to the composition of the gut microbiome (4). Advanced techniques that identify microbial sequences, including 16S rRNA gene and shotgun metagenomic analysis, have provided new insights into the diversity of microbial organisms present both in the diseased and normal gut (5). The composition of the gut microbiome is stable within individuals and largely shared between healthy individuals (1, 6). Microbial imbalance or dysbiosis in the gut microbiome is associated with illness and disorders, including interstitial lung diseases (ILDs) (7–10).

For many years, the lung was thought to be a sterile environment (11). However, recent advances in microbial sequencing techniques suggest that a variety of microbial organisms dwell in both the upper and lower respiratory tract and that composition of this microbial community is altered in respiratory disease states (12). Characterizing the composition of the lung microbiome during disease and elucidating the contribution of the dysbiotic microbiome to disease progression is an area of active research for many respiratory ailments, including ILDs.

ILDs, otherwise called diffuse parenchymal lung diseases, are a group of disorders characterized by chronic inflammation that result in fibrosis (scarring) of the lung (13). The most common symptom of all ILDs is shortness of breath or dyspnea, often accompanied by a dry cough, chest discomfort, fatigue, and occasionally weight loss. Examples of ILDs include sarcoidosis, asbestosis, hypersensitivity pneumonitis, idiopathic pulmonary fibrosis (IPF), non-specific interstitial pneumonia (NSIP), and acute interstitial pneumonitis (14, 15). Some of the risk factors of ILDs are genetics; exposure to hazardous material, such as asbestos; prior infection with microorganisms, including tuberculosis and hepatitis C; radiation and chemotherapy treatments; smoking; connective tissue diseases; and chronic inflammatory diseases like rheumatoid arthritis (16). In some cases, such as IPF and sarcoidosis, the causes may be unknown. There is a growing body of literature supporting dysbiosis of the microbiome as a contributor to ILD, which will be discussed in this review.

Epidemiological and experimental evidence highlights an important cross-talk between the gut microbiota and the lungs (9). Alterations in the microbial community due to factors such as drugs, disease, or diet is linked with changes in immune responses and disruption of homeostasis in the airways, as well as in the gastrointestinal tract (17–20). The altered inflammatory state resulting from microbial dysbiosis, as well as the microbes themselves, may play an important role in ILD progression. Alterations to the microbiome may serve as biomarkers for disease, explain disease progression or serve as a therapeutic target. In this review, we discuss the current understanding of the involvement of the both the lung and gut microbiome in ILD.

## Lung Microbiome Impacts Interstitial Lung Disease Progression

Microbial communities present in a specific body niche will have an impact on the physiology of that body site through direct interactions with host cells. Numerous studies have implicated an altered lung microbiome in the pathogenesis of interstitial lung diseases, such as idiopathic pulmonary fibrosis (IPF) and sarcoidosis. The findings of these studies are summarized in Table 1, and discussed in the following section.

**Table 1:** Studies conducted on microbiome in interstitial lung diseases.

**Disease state**	**Assessment method(s)**	**Conclusions**	**References**
Idiopathic pulmonary fibrosis (IPF)	16S rRNA sequencing of BALF; 454 pyrosequencing; quantitative BALF culture	IPF patients have increased bacterial burdens in lungs, enriched for pathogenic genera like *Staphylococcus* and *Streptococcus*	(22–26)
Sarcoidosis	16S rRNA sequencing of BALF, lymph node biopsies, spleen tissue; shotgun metagenomic sequencing of BALF	Alteration of microbiome in sarcoidosis is unclear—results vary by study and specimen type, but possible loss of diversity in sarcoidosis lung microbial community	(28–31)
Idiopathic interstitial pneumonia (IIP)	16S rRNA sequencing of BALF	No change between lung microbiome of health and IIP patients	(29)
Systemic Sclerosis (SSc)	16S rRNA sequencing of SSc patient fecal samples	Dysbiosis of intestinal microbiome in SSc patients compared to healthy controls	(34)
Silicosis	16S rRNA sequencing of silicosis patient fecal samples	Decrease in microbial diversity in intestinal community with enrichment of Proteobacteria	(8)
Hypersensitivity pneumonitis	16S rRNA sequencing of fecal samples using a murine model of HP	*Bacteroidetes* phylum enriched in streptomycin treated animals that develop severe HP	(36)

### IPF

The lung microbiome of IPF patients is distinct from healthy individuals (21). In one study, IPF patients had a higher lung bacterial load in their lungs enriched for *Haemophilus, Streptococcus, Neisseiria*, and *Veillonella* genera compared to healthy individuals (22). Additional analysis of IPF patient bronchoalveolar lavage fluid (BALF) by 16S rRNA sequencing showed that lung bacteria may play a causative role in acute exacerbation of IPF, and a high bacterial load at the time of diagnosis may be a biomarker for rapidly progressive disease (23, 24). A multicenter cohort study was conducted on the microbial signatures associated with progression of IPF, in which the researchers retrospectively sequenced lung microbiota using 454 pyrosequencing in 55 IPF patients' baseline BALF samples and reported that the presence of specific *Streptococcus* or *Staphylococcus* species above a certain threshold was associated with a faster-progressing disease (25). A different investigation using 16S rRNA sequencing identified the most prevalent operational taxonomic units (OTUs) in IPF patients BALF as *Prevotella* and *Staphylococcus* species (26). Overall, these investigations demonstrate that IPF patients have high bacterial loads in their lungs compared to healthy controls, which are enriched for potentially pathogenic genera such as *Staphylococcus* and *Streptococcus*, implicating the lung microbiome in disease progression.

### Sarcoidosis

Sarcoidosis is an additional ILD of unknown etiology that may be influenced by lung microbiota composition (27). In a cross-sectional study to compare the lung microbiota from bronchoalveolar lavage fluid (BALF) of 71 patients with sarcoidosis and 10 healthy controls, the investigators identified *Atopobium* and *Fusobacterium* as increased in abundance in sarcoidosis samples compared with those from healthy controls using 16S rRNA sequencing (28). In contrast, a different study used 16S rRNA gene-based pyrosequencing to characterize microbial communities in upper and lower airways in ILD patients, including idiopathic interstitial pneumonia and sarcoidosis. The microbiota in lower airways of the majority of patients primarily consisted of *Prevotellaceae, Streptococcaceae*, and *Acidaminococcaceae*. However, according to this study, the diagnosis of ILD did not alter the overall lung microbiota compared to healthy controls (29). Metagenomic sequencing to analyze various specimens including lymph node biopsies, BALF, Kveim reagent samples, and fresh granulomatous spleens from 93 sarcoidosis patients and 72 control subjects revealed elevated levels of certain bacterial and fungal orders in single sarcoidosis sample types but did not detect enrichment of the same orders across multiple sample types (30). Another study investigating microbial composition during airway abnormalities using 16S rRNA sequencing found that the microbiome composition in BALF of sarcoidosis patients is similar to that in rheumatoid arthritis (RA) patients and distinct from healthy controls, with reduced presence of *Actynomyces* and *Burkhordelia*, but enrichment of periodontopathic taxa, including *Treponema, Prevotella*, and *Porphyromonas* (31).

Using these studies and others to develop a consensus “ILD respiratory microbiome,” in addition to optimizing and standardizing a method of sample collection and detection for respiratory microbiome sequencing, could lead to improvements in the diagnosis of ILDs. Missing from the current ILD lung microbiome literature is a dissection of the mechanism by which the genera enriched in the ILD respiratory microbiome impact lung health. Whether ILD pathophysiology drives microbial community changes or the microbial dysbiosis drives ILD progression is also an outstanding question that is key to understanding and treating ILDs.

## Gut Microbiome Impacts Lung Health

**Figure 1:**
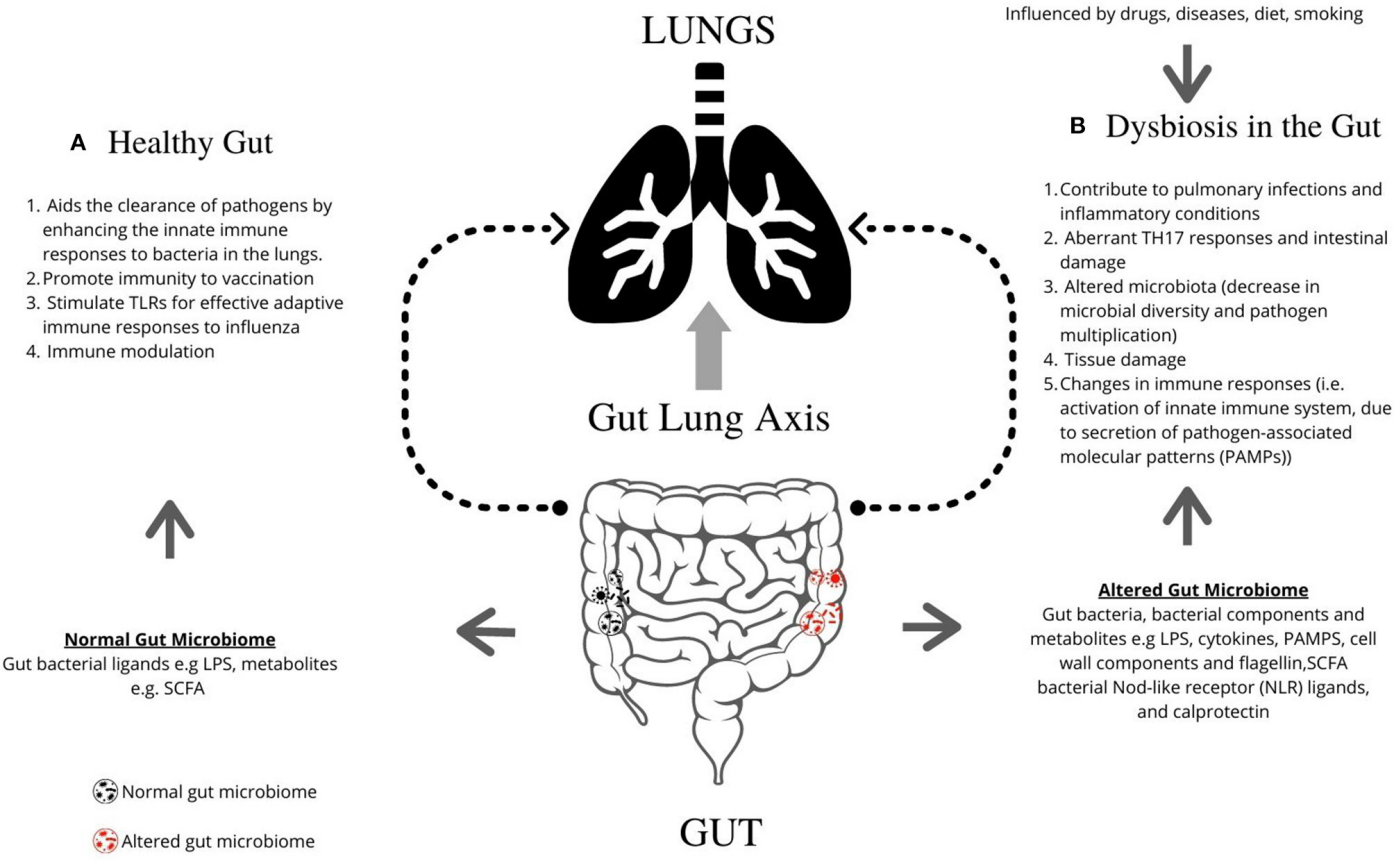
Graphical representation of the role of gut microbiome in regulating lung pathogenesis. The gut microbiome profoundly impacts human physiology and nutrition, and is essential for human life. **(A)** The healthy gut aids the clearance of pathogens by enhancing the innate immune responses to bacteria in the lungs, promotes immunity following vaccination, and can stimulate TLRs for effective adaptive immune responses to influenza. **(B)** Microbial imbalance or dysbiosis in the gut microbiome is influenced by drugs, diet and diseases, and can be associated with illness and disorders, including interstitial lung diseases. Gut bacteria, bacterial components, and metabolites such as LPS, PAMPs, cell wall components, and flagellin contribute lead to changes in lung immunity. Dysbiosis contributes to pulmonary infections and inflammatory conditions, aberrant TH17 responses and intestinal damage, altered microbiota (decrease in microbial diversity and pathogen multiplication), tissue damage, and changes in immune responses [i.e., activation of innate immune system, due to secretion of pathogen-associated molecular patterns (PAMPs)]. TLR, Toll-like receptors; LPS, Lipopolysaccharide; PAMPs, pathogen-associated molecular patterns; SCFA, short-chain fatty acids; NLR, Nod-like receptor.

The respiratory microbiome develops alongside the gut microbiome during early life (32). Epidemiological and experimental evidence highlights an important cross-talk between the gut microbiota and the lungs, called the gut–lung axis. Recent studies suggest that dysbiosis in the gut microbiota is linked with changes in immune responses and homeostasis in the airways in diverse lung pathologies, including ILD, asthma, and pneumonia. Figure 1 describes the mechanisms by which the gut is known to impact lung physiology.

### Systemic Sclerosis

Systemic sclerosis (SSc), also called scleroderma, is an immune-mediated rheumatic disease that is characterized by vasculopathy, fibrosis of the skin and internal organs, such as the gastrointestinal (GI) tract and the lungs, and often progresses to ILD (33). Dysbiotic gut microbiota has been shown in patients with SSc, particularly those with extra-intestinal manifestations, including lung fibrosis (34). In a study that analyzed the link between gut inflammation and ILD in a large SSc population, increased fecal calprotectin levels correlated with ILD progression (35). While this suggests intestinal inflammation and alteration of the gut microbiome are present in ILD, further studies are required to elucidate the role calprotectin and gut dysbiosis may play in the development of ILD in SSc.

### Silicosis

Investigation of the gut microbial composition of fecal samples from 18 patients with silicosis, an ILD caused by inhalation of silica, and 21 healthy subjects using 16S rRNA gene sequencing noted striking changes in the microbial composition. Compared with the healthy subjects, the bacterial diversity of the intestinal microbiome was reduced in patients with silicosis. This decrease in diversity was accompanied by an expansion of Proteobacteria (8).

### Hypersensitivity Pneumonitis

Agents, such as antibiotics, can induce changes in the gut microbiota which alter disease phenotypes in ILDs. For example, a study conducted to assess the effects of antibiotic treatment on a T_H_1/T_H_17-mediated ILD, hypersensitivity pneumonitis (HP), found that the severity of HP was unaffected by vancomycin, but increased dramatically after streptomycin treatment (36). Treatment with either antibiotic altered the gut microbiome of the animals, with Bacteroidetes enriched after streptomycin treatment, while vancomycin treatment led to higher levels of Firmicutes in the intestinal communities.

While these studies using SSc and silicosis patient cohorts and a mouse model of HP indicate there is a connection between the gut microbiome and ILDs, future studies that expand the breath of ILDs investigated for their gut microbiota-dependence and better define the alterations that occur in specific disease states are needed. These studies should include both patient cohorts and studies that model and monitor disease progression in mice, including germ-free mice, to strengthen the claim that the gut microbiome impacts ILD progression or development.

Despite the scarcity of data on the influence of the microbiome on ILDs, there are studies on other inflammatory lung conditions that support the importance of a healthy gut microbiome to promote lung health. Two common non-ILD lung diseases, asthma and pneumonia, are reviewed in the next sections to provide additional evidence for a gut-lung axis and suggest the gut microbiome is a major contributor to lung health.

### Asthma

Asthma is a chronic lung disease where alterations in the gut microbiome have been shown to impact disease progression (37). Reductions in the genus *Bifidobacteria* and increase in *Clostridia* in the intestine are associated with asthma in early life (38). In murine studies, depletion of certain members of the gut microbiome through antibiotic administration early in life enhances future susceptibility to allergic asthma, increasing predisposition to airway diseases and pulmonary viral infections (39). Additionally, neonatal mice administered vancomycin demonstrate an increase in allergic asthma and significant alterations to their gut microbiota as compared to control mice (40).

### Pneumonia

A study focused on the role of the gut microbiota in bacterial pneumonia found that the intestinal microbiota act as a protective mediator during pneumococcal pneumonia (41). This study was carried out by intranasal infection of conventional and germ-free mice with *S. pneumoniae*, and identified that the gut microbiota enhanced primary alveolar macrophage function. Subsequent studies demonstrated that this enhanced macrophage function is mediated through GM-CSF signaling (42) and that the presence of filamentous segmented bacteria protects against *Staphylococcus aureus* pneumonia through induction of type 17 immunity (43).

These studies indicate that the gut microbiota is important for lung health. Interestingly, specific community members are associated with protection from asthma or pneumonia, while others exacerbate disease. This suggests that while future work is needed to uncover the role of specific bacterial species, probiotic or microbiota transfer methods may be successful methods of treating lung diseases.

## Gut Microbiome Impacts Immune Responses

Several mechanisms have been proposed to describe the impact of the gut microbiota on lung physiology. A prevailing hypothesis is that changes in gut microbial composition impact host immune responses in the lung. Support for this hypothesis demonstrating that the gut microbiome impacts immunity in the gut, lungs, and systemically, is discussed in the following section.

### Immunity in the Gut

The gut microbiota is now recognized as an influential player in host immune responses (44, 45). Murine studies show that the gut microbiome, particularly commensal bacteria, are important in shaping the host immune system (46–48). The T_h_17: T_reg_ balance in the lamina propria is determined by gut microbiota composition and specific members of the gut bacterial community may influence intestinal immunity, tolerance, and susceptibility to inflammatory bowel diseases (49). For instance, the segmented filamentous bacteria (SFB), can induce the appearance of CD4^+^ T helper cells that produce T_h_17 cells in the lamina propria. Colonization of the small intestine with commensal SFB resulted in enhanced resistance to the intestinal pathogen *Citrobacter rodentium*, and is linked with an increase in expression of genes associated with antimicrobial defenses and inflammation (50).

### Immunity in the Lungs

The gut microbiota can aid the clearance of pathogens by enhancing the innate immune responses to pathogens in the lungs. In a study designed to investigate the role of commensal microflora in the gut on the host defense in pneumonia through toll-like receptors (TLRs), the authors found that antibiotic pretreatment to deplete commensal gut flora prior to *Escherichia coli* pneumonia challenge led to increased bacterial burdens in both the blood and the lungs. They also noted cytokine suppression (TNF-α, IL-6, IL-1β) as well as suppressed nuclear factor κB activity in the intestine. They concluded that the gut microbiota is critical in inducing TLR4 expression and nuclear factor κB activation of intestinal and lung innate immune defense against *E. coli* pneumonia (51). Bacterial Nod-like receptor (NLR) ligands including a NOD1 ligand, MurNAcTri_DAP_, and a NOD2 ligand, muramyl dipeptide, from the gastrointestinal tract have also been shown to rescue host defenses in the lung (52). Additionally, gut microbiota promote lung immunity following vaccination (53). Cell wall components and flagellin of gut bacteria have been shown to stimulate TLRs for effective adaptive immune responses to influenza (54).

Microbes influence host immunity in many ways, including the ability of *Bacteroides* to expand Treg cell populations or skew the TH1/TH2 phenotype in either direction, and suppress host inflammatory responses through production of short chain fatty acids (SCFAs) (55). SCFAs are produced by many enteric bacterial species and act through free fatty acid (FFA) receptors or epigenetic regulation of immune cells to promote broad anti-inflammatory effects (56). SCFA administration is linked to reduced pulmonary pathology following both and viral and bacterial infection in mice (57–59). Overall, these studies suggest that recognition of the gut microbiota, as well as specific bacterial metabolites, primes the immune response to counter microbial challenges in the lung.

### Systemic Immune Responses

Studies have shown that dysbiosis in the gut microbiome may result in the development of systemic inflammatory conditions, such as rheumatoid arthritis and atherosclerosis (60–62). In the case of sarcoidosis, an altered composition of the gut microbiota may activate the innate immune system, promoting the formation of granulomas through the secretion of proinflammatory cytokines, such as IL-6, IL-12, IL-18, and TNF-α (63).

Due to the relationship between host immunity and enteric microorganisms, various strategies have been put in place to target the gut microbiota as a way of managing or preventing chronic inflammatory diseases. These strategies include the use of antimicrobials to deplete or supplement microbial load, changes in diet, or supplementation with live microorganisms. Microbial transplantation has become a useful tool in manipulating the gut microbiome for management or prevention of certain illnesses. In recent times, fecal microbial transplantation (FMT) strategies have been used in a range of infections with encouraging results (64–66). The mechanisms underlying the success of FMT is still an area of active research.

## Lung Microbiome Impacts Gut Microbiome Composition and Health

In addition to the gut microbiome impacting lung health, there is evidence that changes in lung microbial communities impact gut physiology. This suggests that the gut-lung axis is bidirectional and should be considered during investigations of the impact of gut dysbiosis on lung disease.

### Viral

Perturbations to the lung microbial community, such as the introduction of a respiratory virus, influence the gut microbial communities. For example, murine models of pulmonary influenza virus infection increase *Enterobacteriaceae* while reducing *Lactobacilli* and *Lactococci* in the intestinal microbial community (67). In a study designed to access the occurrence of gastroenteritis-like symptoms using a mouse model of respiratory influenza infection, researchers found that influenza infection altered the intestinal microbiota composition, which was mediated by IFN-gamma production from lung-derived CCR9^+^ CD4^+^ T cells recruited into the small intestine. This resulted in aberrant T_h_17 responses and intestinal damage (68). Perturbations to the gut microbiome as a result of respiratory viruses have also been demonstrated in human studies. A recent study using 16S rRNA sequencing of patient fecal samples described reduced diversity in the gut flora of patients hospitalized with COVID-19 and H1N1 influenza. Interestingly, COVID-19 and H1N1 influenza patients had different bacterial genera that predominated their dysbiotic gut communities (69).

### Bacterial

Pulmonary microbial dysbiosis following intratracheal lipopolysaccharide (LPS) administration in mice is accompanied by disturbances in their gut microbiota secondary to movement of bacteria from their lung into the bloodstream. This causes an increase in the bacterial load in the intestines, thereby disrupting the gut microbial community (70).

Limited, but compelling evidence suggests that the lung microbiota may be influencing the composition of the gut microbiome and overall gut health. These studies address an additional, novel aspect of the gut-lung axis and may help explain the concordance of respiratory and GI symptoms during infection with pulmonary pathogens, such as *Legionella pneumophila* and SARS-CoV-2 (71, 72). Additional studies focused on a variety of states of lung dysbiosis, including ILDs, are necessary to elucidate the impact the lung microbiota has on the gut.

## Conclusions

The impact of the microbiome on human physiology is substantial. Studies evaluating the composition of the lung and the gut microbiome in patients with interstitial lung diseases suggests that dysbiosis in the communities of either body site are correlated with disease. Whether this is true in all types of ILD, and whether the altered community contributes to ILD progression remains to be elucidated. Patient data and mouse models in other lung diseases, such as asthma and pneumonia, suggest that the gut microbiome has a profound influence on lung health. Specific members of the gut and lung communities can either ameliorate or exacerbate disease, indicating further research is needed to define the impact of individual microbes on disease states, including ILDs. Enhancement of the immune response is likely the mechanism by which the gut microbiome is capable of impacting lung homeostasis, as considerable evidence has shown that recognition of gut flora is key to regulating immune responses. Targeting the gut microbiome is an attractive target for therapeutic intervention in lung diseases, but more research is needed to identify the specific alterations that would be beneficial. The literature reviewed here provides support for a link between the microbiome and ILDs.

## Author Contributions

OC wrote the manuscript. WD, LH, and AC modified the manuscript and made final corrections. All authors contributed to and approved the final version of the manuscript for publication.

## Conflict of Interest

The authors declare that the research was conducted in the absence of any commercial or financial relationships that could be construed as a potential conflict of interest.
